# Supplementary material for: Spontaneous conversion of O-tosylates of 2-(piperazin-1-yl)ethanols into chlorides during classical tosylation procedure
Source: R Soc Open Sci. 2019 Feb 13;6(2):181840. doi: 10.1098/rsos.181840 (PMC6408397; doi:10.1098/rsos.181840)
Supplement: Supporting Information [file rsos181840supp1.docx]

**Supporting Information**

Spontaneous Conversion of *O*-Tosylates of 2-(Piperazin-1-yl)ethanols into Chlorides during Classical Tosylation Procedure

Vanya B. Kurteva,*^,†^ Boris L. Shivachev,^‡^ and Rositsa P. Nikolova^‡^

*^†^Institute of Organic Chemistry with Centre of Phytochemistry, Bulgarian Academy of Sciences, Acad. G. Bonchev street, bl. 9, 1113 Sofia, Bulgaria; E-mail: vkurteva@orgchm.bas.bg*

*^‡^Institute of Mineralogy and Crystallography “Acad. Ivan Kostov”, Bulgarian Academy of Sciences, Acad. G. Bonchev street, bl. 107, 1113 Sofia, Bulgaria*

**Table of Contents**

**Figure S1**.^1^H NMR spectra of pirlindole derivatives **4a** (brown) and **5** (green) 2

**Figure S2**.^13^C NMR spectra of pirlindole derivatives **4a** (brown) and **5** (green) 2

**Figure S3.** Partial ^13^C NMR spectra of crude reaction mixture of the tosytation of **2a**

within 30 min (down), **2a**, **4a**, and **3d** 3

**Figure S4**. Partial ^1^H NMR spectra of crude reaction mixtures of **4a** synthesis within

variable reaction times 3

**Figure S5**. Partial ^1^H NMR spectra of **3c** (down), **4c** (up) and crude reaction mixtures

within variable reaction time 4

**Figure S6.** ^1^H NMR spectrum of the crude reaction mixture after reaction between **2g**

and tosyl chloride in pyridine. 4

APPENDIX 5

NMR spectra of ester derivative of **1** 5

NMR spectra of **2a** 6

NMR spectra of **4a** 9

NMR spectra of **5** 12

NMR spectra of **4b** 15

NMR spectra of **2c** 17

NMR spectra of **4c** 19

NMR spectra of **2d** 21

NMR spectra of **4d** 22

NMR spectra of **2e** 24

NMR spectra of **3f** 25

NMR spectra of **4f** 28

NMR spectra of ester precursor of **2g** 31

NMR spectra of **2g** 33

**Figure S1.** ^1^H NMR spectra of pirlindole derivatives **4a** (brown) and **5** (green).

**Figure S2.** ^13^C NMR spectra of pirlindole derivatives **4a** (brown) and **5** (green).


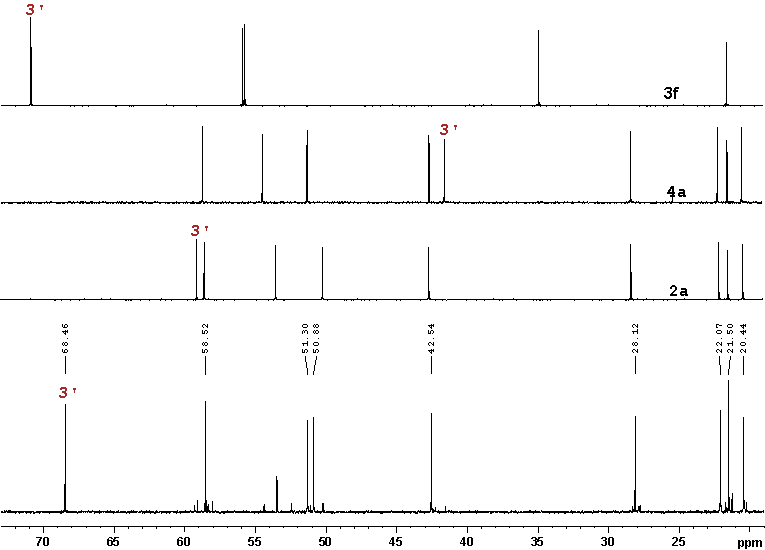


**Figure S3.** Partial ^13^C NMR spectra of the crude reaction mixture of the tosytation of **2a** within 30 min (down), **2a**, **4a**, and **3f**.

**Figure S4.** Partial ^1^H NMR spectra of crude reaction mixtures of **4a** synthesis within variable reaction times.

**Figure S5.** Partial ^1^H NMR spectra of **3f** (down), **4f** (up) and crude reaction mixtures within variable reaction time.

**Figure S6.** ^1^H NMR spectrum of the crude reaction mixture after reaction between **2g** and tosyl chloride in pyridine.The asterisks indicate a part of **3g** signals.

**APPENDIX**

***NMR spectra of crude ester derivative of 1.***

******

******

******

***NMR spectra of 2a.***

***NMR spectra of 4a.***

***NMR spectra of 5.***

***NMR spectra of 4b.***

***NMR spectra of 2c.***

******

******

******

******

***NMR spectra of 4c.***

******

******

******

******

***NMR spectra of 2d.***

***NMR spectra of 4d.***

***NMR spectra of 2e.***

***NMR spectra of 3f.***

***NMR spectra of 4f.***

******

******

******

******

******

******

***NMR spectra of ester precursor of 2g.***

***NMR spectra of 2g.***
